# Reappraisal of Atrial fibrillation: interaction between hyperCoagulability, Electrical remodelling and Vascular destabilisation in the progression of AF (RACE V) Tissue Bank Project: study design

**DOI:** 10.1007/s12471-021-01538-x

**Published:** 2021-01-27

**Authors:** M. D. Gilbers, E. Bidar, B. Maesen, S. Zeemering, A. Isaacs, H. Crijns, I. van Gelder, M. Rienstra, S. Verheule, J. Maessen, M. Stoll, U. Schotten

**Affiliations:** 1 Department of Cardiothoracic Surgery, Maastricht University Medical Centre, Maastricht, The Netherlands; 2 Cardiovascular Research Institute Maastricht, University of Maastricht, Maastricht, The Netherlands; 3 Department of Physiology, University of Maastricht, Maastricht, The Netherlands; 4 Institute of Human Genetics, University of Münster, Münster, Germany; 5 Department of Biochemistry, Genetic Epidemiology and Statistical Genetics, University of Maastricht, Maastricht, The Netherlands; 6 Department of Cardiology, Maastricht University Medical Centre, Maastricht, The Netherlands; 7 Department of Cardiology, University of Groningen, Groningen, The Netherlands

**Keywords:** Atrial fibrillation, Postoperative atrial fibrillation, Translational research, Cardiac tissue, Study design

## Abstract

**Background:**

The development of atrial fibrillation (AF) is a complex multifactorial process. Over the past few decades, much has been learned about the pathophysiological processes that can lead to AF from a variety of specific disease models in animals. However, our ability to recognise these disease processes in AF patients is still limited, which has contributed to the limited progress in improving rhythm control in AF.

**Aims/objectives:**

We believe that a better understanding and detection of the individual pathophysiological mechanisms underlying AF is a prerequisite for developing patient-tailored therapies. The RACE V Tissue Bank Project will contribute to the unravelling of the main molecular mechanisms of AF by studying histology and genome-wide RNA expression profiles and combining this information with detailed phenotyping of patients undergoing cardiac surgery.

**Methods:**

As more and more evidence suggests that AF may occur not only during the first days but also during the months and years after surgery, we will systematically study the incidence of AF during the first years after cardiac surgery in patients with or without a history of AF. Both the overall AF burden as well as the pattern of AF episodes will be studied. Lastly, we will study the association between the major molecular mechanisms and the clinical presentation of the patients, including the incidence and pattern of AF during the follow-up period.

**Conclusion:**

The RACE V Tissue Bank Project combines deep phenotyping of patients undergoing cardiac surgery, including rhythm follow-up, analysis of molecular mechanisms, histological analysis and genome-wide RNA sequencing. This approach will provide detailed insights into the main pathological alterations associated with AF in atrial tissue and thereby contribute to the development of individualised, mechanistically informed patient-tailored treatment for AF.

## Introduction

Atrial fibrillation (AF) is the most common cardiac arrhythmia in adults and associated with a two- to threefold increased risk of cardiovascular events. The overall lifetime incidence ranges between 11 and 45% [1, 2]. There is a clear association between aging and AF, with a 12-fold increase of AF incidence from an age below 60 to above 80 years [3]. As AF is associated with an increased risk for stroke and worsening of heart failure, it represents a significant public health burden [4].

Both acute and chronic pathophysiological mechanisms contribute to the initiation and maintenance of AF. Cardiac surgery can represent an acute factor eliciting AF. With incidences ranging from 15 to 60%, AF occurring during the first postoperative days (early postoperative AF, POAF) is the most common complication after cardiac surgery. POAF often compromises atrial and ventricular pump function, causing prolonged hospitalisation and potentially increased thromboembolic risk [5].

Experimental and clinical studies have demonstrated that a wide variety of pathophysiological mechanisms can cause AF. Clinical conditions such as hypertension and congestive heart failure are associated with a higher incidence of AF. Pathophysiological alterations caused by these clinical conditions include cellular hypertrophy, ion channel remodelling, fatty infiltration and fibrosis, all of which can contribute to the progressive increase in AF susceptibility [6]. In addition to these mechanisms, an individual’s genetic background contributes to a predisposition to AF [7]. Exploring the principal molecular mechanisms leading to AF in patients undergoing cardiac surgery may provide insights into the pathophysiology of the arrhythmia in general [8].

The fact that progress in prevention and treatment of AF has been limited over the past few decades may partly be explained by the broad diversity of underlying mechanisms at the molecular, cellular and organ level. Animal models assessing the effects of cardiovascular comorbidities such as heart failure, hypertension and obesity have led to a better understanding of a large variety of electrophysiological and molecular mechanisms of AF perpetuation. However, techniques to link this knowledge to clinical signs and symptoms, enabling the identification of individual disease mechanisms in patients, have only been developed to a very limited extent. This apparent translational gap represents one of the most important challenges in translational electrophysiology.

The Reappraisal of Atrial fibrillation: interaction between hyperCoagulability, Electrical remodelling, and Vascular destabilisation in the progression of AF (RACE V) consortium is a national collaboration between the University Medical Centres of Groningen, Leiden and Maastricht in the Netherlands. The programme is funded by the Netherlands Heart Foundation and has the goal of identifying regulatory signalling pathways, networks and changes in gene expression which may be held responsible for AF progression with a focus on hypercoagulability [9].

The Tissue Bank Project is a work package of the RACE V consortium and aims to systematically quantify AF episodes during a 2.5-year period following cardiac surgery and to investigate the relationship between pathophysiological changes in atrial tissue samples and the clinical presentation of the patients. Here, we describe the rationale, design, methodology and expected results of this study.

## Objectives

Our primary aim is to study the major molecular mechanisms of AF in atrial tissue samples by combining histology, biochemical analysis and genome-wide RNA expression profiles. Secondly, the incidence of AF during the first years after cardiac surgery in patients with or without a history of AF will be studied. Both the overall AF burden as well as the pattern of AF episodes will be investigated. Finally, we will examine the associations between molecular mechanisms, the rhythm history of the patients and—importantly—the incidence and pattern of AF during the follow-up period.

## Study design

### Patient population and overview

Patients undergoing any type of cardiac surgery, with either an open-chest or thoracoscopic approach, are recruited. Patients with a history of cardiac surgery or surgical ablation for cardiac arrhythmias, younger than 18 years, or with diseases limiting their life expectancy to less than 2.5 years are excluded from the study. We aim to include 200 patients, with a distribution of 100 patients without a history of AF, 50 patients with known paroxysmal AF (PAF), and 50 patients with known persistent AF. Tab. 1 shows all the data gathered during this study.

**Table 1:** Overview of all data gathered during our study. Preoperatively (*T*_*preOR*_), peroperatively (*T* = 0), follow-up visits after 1 (*T* − 1) and 2.5 (*T* = 2.5) years

	T_preOR_	T = 0	T = 1	T = 2.5
Clinical history	X		X	X
Physical examination	X		X	X
Extended digitised electrocardiogram	X		X	X
Blood biomarkers	X			X
Echocardiography	X			X
Vascular assessment			X	
Questionnaires	X		X	X
Low-dose cardiac CT	X			
Cardiac biopsies		X		
Rhythm monitoring				

### Sample size

**Fig. 1:**
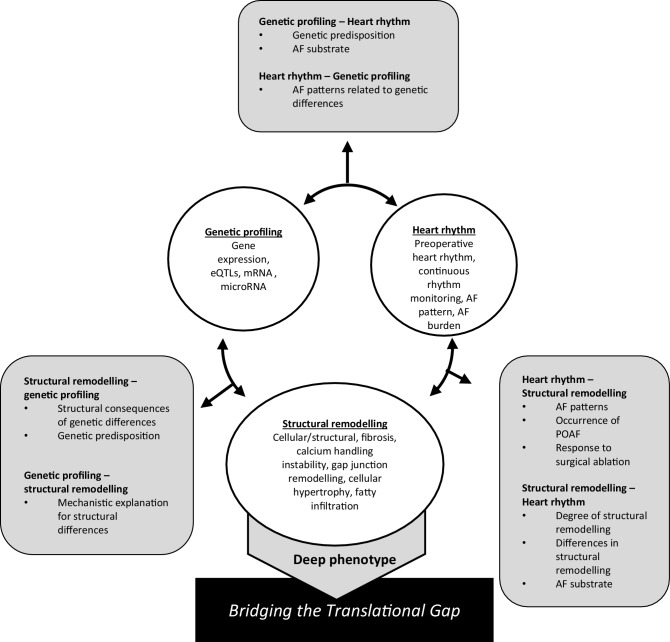
After combining an extensive clinical background with non-invasive measurements and cardiac tissue, we will look for: (1) the combination of genetic data of blood and tissue in combination with data on structural remodelling, which would give insight into the different genes involved in structural remodelling; (2) the possible association or predisposing role of structural remodelling in atrial fibrillation (*AF*) and AF development; (3) the difference in genetic expression in association with different types of AF and the genetic markers for predisposition for postoperative atrial fibrillation (*POAF*) development. *eQTLs* expression quantitative trait loci, *mRNA* messenger RNA

Power calculations were performed in R utilising the RNASeqPower package (Terry Therneau, Steven Hart and Jean-Pierre Kocher [2019]. Calculating sample size estimates for RNA Seq studies. R package version 1.22.1.). These analyses, performed with an extremely stringent *p*-value threshold (*p* ≤ 2.5 × 10^6^), showed that an atrial fibrillation group of 50 patients in comparison to a sinus rhythm group of 100 achieved greater than 80% power to detect a fold change ≤ 1.75 even for rarely expressed transcripts and nearly 100% power for larger effects (see Fig. 1). For comparisons between two patient groups comprising 50 patients each, 80% power was reached for fold changes ≤ 1.75 for more abundantly transcribed genes, and close to 100% power was observed for larger effects irrespective of transcript abundance. These effect sizes are in line with previous observations in the literature.

### Preoperative patient characterisation

After inclusion, in addition to a clinical history, physical examination and questionnaires (Toronto AF Severity Scale, EQ-5D-5L), an extended digital electrocardiogram (ECG) recording (12 standard + 3 additional leads, duration 5 min), transthoracic echocardiography (TTE) and a cardiac computed tomography (CT) scan will be performed. Furthermore, blood samples will be taken 1 day before surgery for the determination of biomarkers related to AF and AF progression. Blood samples will also be used for genotyping.

### Peroperative procedure

During surgery, the surgeon performs biopsies to remove tissue samples from the right and left atrium and the left ventricle. The tissue is snap-frozen for storage at −80 °C. During surgery, an implantable loop recorder (ILR) (Reveal Linq, Medtronic, Minneapolis, MN, USA) is placed subcutaneously. Patients with a cardiac implantable electronic device with an atrial lead, compatible with Carelink Network (Medtronic), will not receive an ILR since continuous rhythm monitoring is already possible via a standard read-out of the device.

### Postoperative follow-up

During the follow-up period of 2.5 years, continuous rhythm monitoring will be performed via the Carelink Network. Patients will be invited to return for follow-up visits after 1 year and 2.5 years postoperatively.

At the 1‑year follow up, clinical history, questionnaires and digital ECG will be repeated. To assess the vascular status of the patients, intima-media thickness in the carotid artery and pulse wave velocity for vascular stiffness will be measured.

At the 2.5-year follow up, clinical history, digital ECG, questionnaires, TTE and blood sampling will be repeated. At the request of the patient, the ILR will be extracted.

## Detailed description of patient characterisation

### Continuous rhythm monitoring

Continuous remote monitoring is available via the Carelink Network. Every night, an automatic read-out of the implanted device data is transmitted, and once a week, a full report of the arrhythmias and conduction abnormalities is acquired. This information gives insight into newly developed AF, recurrences, patterns and burden of AF.

### Extended ECG recordings

Electrocardiography provides insights into electrical propagation in the atria and ventricles and, therefore, contains information on the electrical and structural remodelling of the heart, leading to alterations of electrical impulse conduction [10]. The 12-lead ECG is the golden standard for AF diagnosis, but more detailed investigation using more leads and longer recordings might also reveal information on the existence of an AF substrate [11].

Electrocardiographic parameters determined from the extended digital 15-lead ECG recording, dominant atrial fibrillation frequency, signal-averaged P‑wave duration, dispersion, morphology and spatiotemporal parameter variability, will be correlated to the clinical, genetic and histological properties in our patient population.

### Imaging

Several imaging modalities have been shown to be useful in identifying risk factors that contribute to an arrhythmogenic substrate. For this reason, vascular measurements, a low-dose cardiac CT scan without contrast and TTE will be performed in all patients.

### Biomarkers for AF

Blood sampling will be performed preoperatively and repeated at the 2.5-year follow-up visit. We grouped biomarkers of interest as related to myocardial injury, inflammation, calcium handling, vascular destabilisation, fibrosis and hypercoagulability [9].

### Cardiac tissue characterisation

#### Atrial biopsies

Atrial remodelling is both a cause and a consequence of AF [12, 13]. Histological examination in animals and humans has shown diverse structural alterations linked to AF. To investigate different aspects of structural alterations, we will combine both histological and immunohistochemical staining in cryosections.

#### RNA sequencing

RNA sequencing will be used to determine genome-wide expression profiles in all atrial tissue samples. This hypothesis-generating approach will allow us to identify differentially expressed transcripts in patients with and without AF and also in patients who develop AF after follow-up. The design will, therefore, allow us to determine not only how expression changes as a consequence of AF but also which expression patterns are associated with the development of AF after performing the biopsies.

## Expected results

By combining continuous rhythm monitoring with in-depth molecular-to-clinical phenotyping of patients—fed by preclinical proof-of-concept studies—clinical and biological mechanisms responsible for AF progression can be unravelled. The main strength of this study will be the combination of a thorough clinical phenotype combined with cardiac tissue analysis, long-term continuous rhythm follow-up and blood biomarkers. We expect that this approach will facilitate a broader appraisal of histological findings, transcriptional differences, and allow detection of new pathophysiological pathways (Fig. 1).

Heart rhythm monitoring will allow us not only to determine the factors that are associated with AF but also those that are associated with newly developed AF during follow-up. Such factors are likely not the consequence of AF but possibly play a causative role in AF. In contrast, those properties identified in patients with AF could either be a cause or consequence of AF. This principle can be applied to any clinical, histological or transcriptional parameters studied in our investigation. In this way, we will be able to identify histological properties associated with the presence of AF but also those that likely increase the chance of developing AF because they were identified in patients who did not have AF during or before biopsy but developed AF later on. Potentially, this approach will result in the identification of new candidate mechanisms underlying the occurrence of AF. In addition, the expression profiles of patients may allow us to link expression to differences in structural remodelling, further explaining the underlying mechanisms responsible for AF.

The data set also has the potential to shed more light on how common gene variants that are associated with AF translate into a phenotype explaining this association. As we will have the genomic information of these patients, we can study how expression differs between patients with and without a common gene variant associated with AF. Such expression quantitative trait locus studies are required to learn more about the role of genetic variants in the occurrence of AF.

This unique combination of all three modalities embedded in a well-defined clinical profile gives us the prospect of narrowing the previously described translational gap, which is imperative for the improvement of therapeutic options. A better perception of underlying mechanisms will improve diagnostic possibilities and, therefore, pave the way for individualised patient-tailored therapy.

### Expected insights from improved rhythm monitoring

The current choice of treatment is generally based on the patient’s symptoms and on the type of AF (paroxysmal versus persistent). For example, PAF seems to respond better to catheter ablation than persistent or longstanding persistent AF [14]. However, the clinical differentiation between these AF patterns is often difficult. In a cohort of 1149 patients equipped with a pacemaker, 1‑year follow-up showed that only around half of the patients with PAF and one-third of the patients with persistent AF were correctly diagnosed [15]. Rhythm monitoring using an ILR will provide an accurate characterisation of the actual AF pattern, leading to a more accurate differentiation between PAF and persistent AF.

The use of continuous rhythm monitoring postoperatively has proven valuable in the detection of previously ignored occurrences and recurrences of POAF after hospitalisation [16]. Similar to the results in this study, we expect that almost half of the patients without a history of AF will either develop POAF in the early phase or experience new AF episodes after discharge. Furthermore, the rate of recurrence of episodes after POAF is anticipated to be high. The characterisation of these previously undetected episodes will help us understand the fundamental disparities and establish possible risk stratification.

### Limitations

Patients undergoing cardiac surgery are not entirely representative of the overall population of AF patients. Patients selected for cardiac surgery present with a complex multimorbidity. In our cohort, we included patients with various indications for cardiac surgery. We are aware that the underlying mechanisms for severe aortic stenosis, mitral valve regurgitation and ischaemic disease can have different effects on the development of AF. However, it is the purpose of this study to investigate the diversity of mechanisms that will also likely reflect the diversity of the cardiac diseases in this cohort.

## Conclusion

The RACE V Tissue Bank Project combines deep phenotyping of patients undergoing cardiac surgery, including rhythm follow-up for 2.5 years with a detailed analysis of the principal molecular mechanisms in these patients as assessed by high-throughput histological analysis und genome-wide RNA sequencing. This approach will provide detailed insights into the prevalence of AF following cardiac surgery and how AF and its comorbidities are linked to molecular mechanisms in the right and left atria. The study will also provide a large amount of information on molecular pathways underlying the main pathological alterations associated with AF, heart failure and other cardiovascular diseases in atrial tissue and thereby contribute to the development of individualised, mechanistically informed patient-tailored treatment for AF.
